# Nature and the mind: Towards nature-based mental health care and prevention

**DOI:** 10.1371/journal.pmen.0000647

**Published:** 2026-08-26

**Authors:** Giulia Vivaldi, Emeka Chukwusa, Nick Bridge, Michael Smythe, Ioannis Bakolis, Lisa Page, Rhiannon Thompson, Matthew White, Andrea Mechelli

**Affiliations:** 1 Department of Health Service & Population Research, Centre for Mental Health Policy & Evaluation, Institute of Psychiatry, Psychology, and Neuroscience, King’s College London, London, United Kingdom; 2 King’s College London, London, United Kingdom; 3 Bethnal Green Nature Reserve Trust, London, United Kingdom; 4 Brighton & Sussex Medical School, Sussex, United Kingdom; 5 Herbert Smith Freehills, London, United Kingdom; 6 Department of Psychosis Studies, Institute of Psychiatry, Psychology, and Neuroscience, King’s College London, London, United Kingdom; PLOS: Public Library of Science, UNITED KINGDOM OF GREAT BRITAIN AND NORTHERN IRELAND

## Abstract

The United Kingdom (UK) Government’s recent 10 Year Health Plan for England aims to fix a “broken” and increasingly unaffordable health system through a shift from sickness to prevention, hospital to community, and analogue to digital, with a focus on mental as well as physical health. However, the 171-page strategy for a healthy nation does not contain a single reference to nature or biodiversity. We review the significant benefits of nature and biodiversity to health and outline how these benefits can and should be incorporated into the 10 Year Health Plan. We provide examples of effective approaches and techniques, describe challenges to implementation, and propose potential solutions. We focus on mental health, although many benefits will also apply to physical health. Harnessing nature and biodiversity could play a substantial role in supporting the UK’s revised goals, particularly preventing illness and enhancing treatment at the community level.

## Introduction

### Background

Humans are a part of Earth’s ecosystem—an integral part of nature alongside millions of interdependent species. Living harmoniously within our ecosystems, particularly within urban centres, is essential to our health and the sustainability of the natural world. Human and planetary health, therefore, are one and the same. That is why the greatest threat to human health globally will come from destabilisation of the climate, degradation of ecosystems, and the disruption of Earth’s life-supporting systems [1].

Yet modern medicine often overlooks our relationship with our physical environment in treating or preventing mental ill-health. Public health bodies in the United Kingdom (UK) raise awareness about environment-related health risks, but there is limited integration with National Health Service (NHS) operations and mental health services. In the UK, the National Climate Information Centre (NCIC) and the UK Health Security Agency’s (UKHSA) newly established Centre for Climate and Health Security are responsible for raising awareness of the public health impact of climate change, developing evidence, and informing climate change policies. However, both have limited influence on the NHS. Additionally, healthcare services are often reactive rather than proactive, limiting their success in addressing environment-related health risks.

In the UK, publication of the government’s 10 Year Health Plan for England [2] offers an opportunity to bridge these gaps. Here, we highlight how this can be achieved by incorporating our natural environments into mental healthcare planning and delivery. For the purposes of this paper, we use the term “nature” broadly to refer to natural and semi-natural environments, including parks, woodlands, rivers, coastlines, urban greenspaces, and other settings that support biodiversity and provide opportunities for contact with the natural world. While this paper focuses primarily on the role of nature in promoting human health and wellbeing, we recognise that this represents a predominantly anthropocentric perspective. Nature also possesses intrinsic value independent of its benefits to humans, and ecological and ethical considerations outside the scope of this article provide important complementary justifications for their protection and restoration. While this article focuses on England, the review of the evidence and the perspectives will be relevant to other countries.

### Rise in mental health problems

The prevalence of Mental health problems, including depression, anxiety and severe mental disorders is rising globally [3], particularly among children and adolescents [4] and mental ill health is now one of the leading causes of disability in the UK, affecting nearly half of working-age disabled adults [5]. At least one in two people are likely to experience mental health problems during their lifetime [6]. In addition, severe mental disorders are associated with a drastic reduction in life expectancy [7]. A systematic review and meta-analysis of 109 studies from 24 countries found that Pooled life expectancy for mental disorders was 63.85 years [8].

Despite the growing prevalence of mental health conditions, physical health has historically been prioritised over mental health [9]. There has been progress on this front, with successive governments calling for greater integration of mental and physical health services and so-called “parity of esteem” [2,9]. However, these commitments have not translated into action, with lengthy waiting times [10], and limited treatment options [11] increasing the risk of chronic conditions. The impact is vast: the financial cost alone of mental ill-health in England is estimated to be at least £300 billion—greater than the entire budget of NHS England [12].

Population-based preventive strategies are therefore needed to improve mental health over the life course for the general population (universal prevention) and those in vulnerable communities (targeted prevention).

### 10 Year health plan for England

Prevention is at the heart of the UK Government’s 10 Year Health Plan for England, which aims to build a health service “fit for the future” [2]. The Plan features three key shifts: from sickness to prevention, hospital to community, and analogue to digital. The move towards prevention is gaining momentum due to societal costs of poor health [13]. Whereas treatment of ill-health is the domain of health and social care systems, prevention is a broader mission. The environmental and social determinants of ill-health are wide ranging; [14,15] thus, solutions to preventing ill-health must also be wide ranging, considering people and their health as part of their environment and working across sectors to achieve the improvements needed.

A key factor for improving population mental health is greater access to green space [16]. The Biophilia hypothesis, that humans have an innate, evolutionary affinity for other living things and connection to nature [17], suggests nature can protect and support mental health. This broad theory provides foundation for more specific frameworks including Attention Restoration Theory [18], which propose that natural environments engage our attention effortlessly and reduce mental fatigue, and Stress reduction theory [19], which suggests that exposure to nature elicits psychological and physiological responses that reduces stress. More recently, additional mechanisms have been identified to explain the benefits of nature for human health. These include changes in the gut–brain–immune axis [20] and the mitigation of the impacts of air pollution [21] and extreme heat [22]. These pathways do not operate in isolation but are likely to interact and reinforce one another [23]. Greater biodiversity has been shown to be associated with reduced risk of anxiety and depression [24] and better mental health outcomes [25]. In those who develop an illness. This suggests that benefits may extend beyond supporting recovery among people with existing mental health problems and may also play a preventive role [26,27].

However, while the Plan acknowledges the importance of factors like housing, transport, and education in preventing ill-health, the contribution of nature to our health and wellbeing is striking in its absence.

### Connecting the dots: Climate change, biodiversity loss, and human health

To make an NHS fit for the future, we need to consider the wider context in which the health system will operate. There is growing awareness of the impacts of climate and environmental change on mental health. For example, higher ambient temperatures are associated with increased suicide incidence [28]. Flooding and wildfires, linked to changes in rainfall and ambient temperatures, can lead to depression, anxiety, and post-traumatic stress disorder [29].

Biodiversity sustains human life through clean air, water, food security, and the many flora, fauna, and fungi underpinning medicine and pharmacology [30], making its loss a threat to our health and wellbeing. Biodiverse environments have benefits for physical and mental health [23], with tree canopies and green spaces reducing the impact of urban heat islands [31]. Conserving and boosting biodiversity is key to mitigating the effects of climate change [32].

Human health is closely tied to ecosystem stability. The reimagining of our health system triggered by the Plan therefore marks an opportunity to acknowledge this relationship and embed it into health policy, recognising that a healthy human society and affordable, functioning health system is partly achieved through an enriched natural environment and biodiversity.

Below, we outline opportunities to incorporate nature and biodiversity into mental healthcare in England, based on the three pillars of the Plan. We provide examples of effective approaches, describe challenges to implementation, and propose solutions—most of which will be applicable to other countries and health systems. Finally, we consider four practical steps to integrate nature into the upcoming reform of the NHS.

## Opportunities and challenges

### From sickness to prevention

Effective prevention of ill-health requires more than healthcare – it depends on education, diets, housing, and environments. It is essential that healthcare reform be integrated with developments in other sectors that can have huge impacts on the nation’s health.

#### Integration of cross-sectoral preventive measures into health policy.

A key example of effective cross-sectoral integration is Scotland’s Natural Health Service programme [33]. The programme involves the collaborative development of action plans with partners across various sectors to achieve two main objectives: increase public awareness of the use and benefits of green health as part of daily life and integrate nature-based health promotion initiatives and structured interventions into routine health and social care policy and practice.

The success of this approach has been illustrated by the four pilot Green Health Partnerships which have shown how improving cross-sectoral collaboration can enable greater use of local green space as a health-promoting resource [34]. In its three-year evaluation, the Green Health Partnerships reported that 548 opportunities for green health activities. A total of 445 awareness-raising and capacity building activities were delivered to health, social care and green health delivery staff, engaging 19,637 individuals, In addition, 63 referral pathways were established by which green activities can be prescribed or recommended, 300 public information and promotion activities were delivered, and the Green Health Partnerships were referenced in 58 local policies and plans. For example, the Green Exercise Partnership established the NHS Greenspace Demonstration Project to show how improvement to NHS outdoor spaces—which comprise over half of NHS Scotland’s estate—could be delivered in practice, while assessing the benefits of this investment for health and wellbeing, biodiversity, and climate change [35]. The programme demonstrates that the mental health benefits of nature can be maximised through collaboration across sectors and integrated planning from the outset recognising the interdependence of human health, social systems, and natural environments.

#### Urban greening and rewilding.

Urban settings require particular attention for preventive measures, as they often struggle with declining biodiversity and higher temperatures due to the urban heat island effect. These challenges are often compounded by an extractive mindset towards nature and biodiversity, which treats the natural environment primarily as a resource to be exploited rather than a system to be protected and restored. Such perspectives can undervalue the broader health, social, and environmental benefits of biodiversity and urban greenspaces. Addressing these challenges requires approaches that recognise the interconnectedness of human wellbeing and ecological systems.

The protection of green spaces should be prioritised to encourage regular interaction with nature among local communities, mitigate extreme heat and air pollution impacts, increase community level food security, and protect biodiversity [36]. Green infrastructure programmes, including London’s Rewilding, Nature Recovery, and Evidence programmes (which is restoring or creating 116 hectares of habitat for wildlife across 22 projects [37], should be designed to interlink responses to the climate and ecological emergencies with tackling disparities in living environments and health outcomes. In practice, this could involve initiatives, e.g., transforming unused land in urban centres into spaces for biodiversity, social connection, and food production. A study by Stanford University’s Natural Capital Project explored how urban farms and food forests in San Antonio, Texas—a city of 1.5 million residents—could transform underutilised land into social and ecological hubs [38]. Their findings suggest such projects in that city alone could provide enough food annually to feed nearly 314,000 households, along with $3.5 million in urban cooling benefits.

The advantages of such initiatives extend beyond food. Food forests can improve community health by increasing biodiversity, expanding access to fresh produce, increasing accessible green space, and providing opportunities for exercise and social connection. They also capture carbon, manage stormwater to reduce flooding, and provide natural cooling. Taken together, these initiatives show how urban greening can be effective to building healthier, resilient cities.

Importantly, development alone of biodiverse green spaces is insufficient to reap health benefits; the benefits from urban green spaces—particularly in new or underserved neighbourhoods—are maximised when the local community engage with these spaces through community-led events [39]. To enable this, barriers to engagement (e.g., safety concerns) and misconceptions (e.g., large green spaces are more beneficial) need to be sensitively tackled with local communities. The Future Parks Accelerator [40], a 3-year programme to support local authorities in improving their green urban spaces, highlighted how nine local authorities addressed these barriers. Among the approaches taken were Camden and Islington’s consultations with voluntary sector partners to understand the needs of underserved communities, Edinburgh’s participatory workshops with residents to determine which projects to run in local green spaces, and Plymouth’s social enterprise approach to support local businesses to start up and grow in green spaces. Potentially benefitting up to five million residents, evidence generated through these initiatives enabled local authorities to secure a combined £43 million in further investment for parks and greenspaces. Other impacts of the Future Park Accelerator programme includes demonstrating the role of urban green spaces as essential public infrastructure, strengthening the evidence base on the social, environmental, and health value of urban green spaces, developing practical guidance and recommendations to improve their accessibility and uptake and positioning green space at the heart of community life [41].

The social implications of urban greening and rewilding should also be considered [42]. Of particular concern is green gentrification, in which environmental improvements to an area can price out vulnerable residents. Urban greening and rewilding programmes should therefore be carried out in consultation with residents; for example, in several US cities, redevelopments are scrutinised by design review boards that include community members and external pro-bono specialists, to ensure that changes to the neighbourhood will benefit existing residents [43]. The Right To Grow movement [44], which requires local authorities to enable community creation of gardens for food and nature on public land, is an example of how local greening can directly serve local residents, with decisions on how the land is used placed in their hands. Additionally, urban greening and rewilding programmes should be developed alongside improved housing policies that provide existing residents with security—for example, community land trusts and cooperative housing models can and have been used to secure housing for community members in areas undergoing greening [43], thus ensuring social and ecological wellbeing. There is a need to make greater use of routinely collected longitudinal data to evaluate the impacts of urban greening and rewilding schemes. Scenario based modelling studies should be conducted to assess the potential impact of urban rewilding programs on local communities and to ensure that such schemes benefit existing residents. Future research should examine the impact of environmental improvements on local demographics, housing affordability, and housing security, generating the evidence needed to inform policies that protect communities from displacement and ensure that the benefits of urban rewilding are shared equitably [45].

#### Bringing nature to young people.

Children and young people, whose futures are impacted by the changing climate, shoulder an increasing mental health burden. A new approach is urgently needed, not just to treat poor mental health in this group, but to prevent problems from ever developing. The young people’s mental health crisis is complex, and effective prevention will require a multi-pronged approach, that addresses factors, such as inequality and deprivation, education, parental and family support, health and social care, access to activities and play, and opportunities for learning, development and growth alongside environmental quality and access to nature. Due to societal shifts towards urbanisation and digital technologies, children are increasingly disconnected from nature, with implications for their physical and mental health [46]. Reconnecting children and young people with the natural world has the benefit of improving their health while fostering environmental awareness. The Our Bright Future program [47], led by The Wildlife Trusts, took a cross-sector approach to this challenge, aiming to engage young people in the environment. More than 128,000 young people participated in the programme, which improved 3000 community spaces and created 350 nature-rich areas. Crucially, 86% of participants reported that taking part had improved their mental health, while 95% reported increased confidence.

However, programmes to bridge the gap between children and nature must consider inequity in green space access, with differences seen by socioeconomic status and ethnicity [48]. Integrating, nature-based activities within educational activities, while simultaneously improving the green spaces near schools, can ensure access to the natural world for children. School-based programmes, like the Children and Nature Programme [49], showcase the work that can be done between the education and environmental sectors to protect biodiversity and improve outcomes. Funding access to growing and cooking programmes like the Hackney School of Food [50] can further build upon this work by giving hands-on experience of food cultivation and land stewardship. The aims of such initiatives stretch beyond supporting mental health and wellbeing, improving school attendance, behaviour, physical health, and care and concern for the environment.

#### Embedding nature in health education.

There has been recent activity on bringing principles of sustainable healthcare into all medical school curricula [51]. In addition, some Royal Colleges have introduced sustainability criteria into their postgraduate curricula and developed networks for practising doctors to support their nature-based practice [52]. However, medical education on planetary health and its connection to human health varies across UK medical schools, with huge disparities in depth of coverage across institutions and training programmes [53]. The students of today are the healthcare leaders of tomorrow. The international student-led initiative The Planetary Health Report Card [54], which evaluates and improves planetary health content in health-professional schools, is a powerful illustration of the momentum and change these future leaders can inspire. Embedding the principles of environmental sustainability and biodiversity protection in all health education pathways, with central guidance and mandated learning outcomes [53], is a key step in ensuring joined-up, long-term thinking within healthcare about our natural environment and the health benefits it can bring. These curricula should be strongly evidence-based, regularly updated in line with the rapid pace of scientific developments in this field, and periodically evaluated to assess their impact on clinical practice. Indeed, developing sustainable and nature-focused leadership within the healthcare system is particularly important [55] while green initiatives—e.g., NHS Forest [56], which strives to improve healthcare green spaces and biodiversity—remain elective rather than mandated.

### From hospital to community

To truly bring health into the community, mental health and wellbeing-supporting measures need to be strengthened in every level of a community, from its local healthcare hubs to the people they serve.

#### Grassroots initiatives.

Grassroot, community-led initiatives such as rewilding of local areas and rivers can have positive effects on the landscape and empower individuals to become active land caretakers, providing social and health co-benefits. Funding for such initiatives largely comes from charities, like Rewilding Britain. Non-profit organisations and wildlife trusts run projects to encourage connection to nature among young people, disadvantaged communities, and mental health service users, among others. Growing Communities, a non-profit organisation that aims to feed urban communities in a fair and sustainable way, shows the impact community-led initiatives can have. Among their projects is Dagenham Farm, the transformation of an ex-council nursery into a growing and learning site [57,58], which is now a self-sufficient organic farm. The farm has hosted various outreach projects, e.g., Grown in Dagenham [58], which taught hundreds of residents how to grow and cook food and offered specific sessions for residents with mental health issues. Such projects and organisations should be encouraged, empowering communities to care for their environments and themselves.

#### Increasing public awareness of nature’s mental health benefits*.*

While many people view nature as important in their lives, there is limited public understanding of the mechanisms through which it supports mental health. Nature exposure can mitigate physiological stress, support cognitive development [59], reduce loneliness [60], anxiety, and depression [61], reduce air pollution and extreme heat and support a healthy gut microbiota [62]. Yet these multiple benefits are not well understood by many. A campaign to help people recognise these benefits within their everyday lives could have significant public health impact, especially at a time of rapid urbanisation and digitisation which are reducing opportunities for direct contact with nature and, amplifying our disconnection from it. This phenomenon has been described as the ‘extinction of experience’, referring to the progressive loss of everyday interactions with the natural world [63].

#### Green social prescribing*.*

Green social prescribing is a way that the NHS already integrates the health of the population with the natural environment outside of the hospital setting. These programmes encourage participation in nature-based interventions, e.g., walking schemes and community gardening projects. The practice can support mental and physical health, promote social inclusion, protect biodiversity through conservation projects and habitat creation.

However, the green social prescribing programme faces various issues [64], including bottlenecks in clinical pathways, inconsistent referral mechanisms, a reliance on short-term funding, and delivery at local level but difficulty embedding it across the healthcare system as a whole. A fragmented and continually shifting provider landscape can create problems for primary-care clinicians attempting to find suitable programmes for their patients, while secondary-care practitioners may not have the same access to established referral routes [65,66]. Green activity providers are often reliant on short-term competitive funding arrangements, disrupting long-term planning and continuity of care, and creating a challenge for healthcare professionals to stay on top of what’s currently available [66]. Social prescribing providers, often based in the voluntary sector, can also lack the training and expertise for managing individuals with complex physical or mental health needs [66].

A holistic and collaborative approach is needed to improve the implementation of green social prescribing. Greater awareness of the practice needs to be built among clinicians [65], with appropriate investment and support provided for practical issues such as identifying providers and referring patients. To support providers and expand provision to existing community gardens, meaningful incentives such as long-term funding and training opportunities should be secured; such steps would improve continuity of access and avoid loss of institutional knowledge. Improved training to support service users with complex needs and increasing the diversity of activities available would improve the inclusivity of the service. Barriers (operational or implementation) to participation need to be removed, by improving transport offerings, expanding to existing community gardens, and developing suitable green spaces in areas which are lacking. Green prescribing policies must successfully be monitored evaluated and implemented to determine whether have achieved their desired outcomes and whether resources were used effectively [65]. The establishment of clear evidence-based protocols would help ensure that green social prescribing interventions are delivered consistently, evaluated rigorously, and scaled effectively across different socioeconomic contexts. Another challenge is the limited awareness and understanding of the benefits of green social prescribing [65]. Addressing this gap requires systematic evaluation of intervention effectiveness using patient-reported outcome measures and standardised assessment tools, such as the Patient Health Questionnaire (PHQ-9), Generalized Anxiety Disorder Scale (GAD-7), and the Warwick–Edinburgh Mental Wellbeing Scale (WEMWBS). These measures would enable more robust assessment of outcomes and generate empirical evidence on the effectiveness of green social prescribing. Such evidence would support more efficient resource allocation, strengthen the case for integration into clinical practice, and inform future policy development.

Implementation science frameworks, outcomes and study designs are essential to understand multilevel barriers, design strategies to overcome those barriers, and analyse the ability of those strategies to advance the uptake and scale-up at the local, national and international arenas [67–69].

### From analogue to digital

The digitalisation of society has been partly blamed for our increasing disconnection from nature and the ‘extinction of experience’. However, digital healthcare also presents opportunities to encourage people back into nature by increasing awareness of nature’s benefits. For example, our team have developed NatureBoost – a smartphone-based 30-day programme to encourage people to engage with nature as part of everyday life [70]. The extent to which technologies such as rewards platforms and mobile applications can foster authentic connections with nature, rather than contributing to further disconnection through increased digital engagement, should be carefully evaluated. Future research should use objective measures to assess how such tools influence nature contact and engagement, ensuring that digital technologies function as a bridge to physical environments rather than a substitute for them.

#### Rewarding people for interactions with nature*.*

The 10 Year Health Plan outlines the creation of a digital health reward scheme, which will reward participants for taking positive actions to improve their health. When developing this scheme, engagement with nature should be incorporated as a positive action participants can take to improve their mental and physical health. The BetterPoints scheme [71], used by several local councils across the UK, encourages green and healthy behaviours by rewarding app users for physical activity and active travel. BetterPoints users are rewarded for time spent doing physical activity, recorded through phone apps and wearables, with points that are exchangeable for vouchers. While focusing mainly on exercise and carbon reduction from active travel, the scheme can be personalised to include specific programmes, e.g., the Love Your Park programme in Ealing [58]. This programme rewarded residents for taking part in park-based community events. Digital incentives could similarly encourage exploration and use of local green spaces, linking to community-led events and reaping the health benefits.

#### Incorporating nature into the NHS app*.*

The NHS app is expected to undergo significant development within the 10 Year Health Plan, converting it to a “full front door to the entire NHS” and giving patients greater control over their health [2]. Key improvement outlined includes tools to advice and signpost patients for non-urgent care; self-referrals; and links to support services, e.g., social enterprises and community groups. The app creates opportunity to bring together several suggestions outlined in this paper. To build public awareness, the benefits of nature exposure could be incorporated into advice given by the app for non-urgent mental health self-care, along with personalised information on local nature-rich environments and events. The app could highlight opportunities for green social prescribing and streamline self-referral to social prescribing link workers or local nature-based initiatives. It can also serve as a platform for rewilding initiatives and support services offered by community-led groups, charities, and wildlife trusts.

In addition, systematic data collection techniques such as the use of smart nature surveillance systems can improve monitoring and tracking over time. Nature surveillance could capitalise on existing infrastructures. For example, in recognition of the need for the NHS to respond dynamically to climate risks, the Greater London Authority and Mayor’s Office for London are implementing a new air quality alert system, targeted specifically at clinicians in General Practices and Emergency Departments [59]. Building on this emerging healthcare system adaptation, nature surveillance and warning systems could be further integrated into NHS operations and incorporated into digital tools to support both patients and clinicians. At the same time, there is a need for further large-scale studies employing robust research methodologies. Effective policy integration requires high-quality longitudinal evidence capable of establishing causal relationships between specific nature-based interventions and health outcomes. Such evidence would provide a strong foundation for the large-scale adoption of nature-based solutions within clinical practice and policy.

### Four practical steps to bring nature into the 10 Year Health Plan

1 *Integrated approach*

By excluding nature from its 10 Year Health Plan, the Department of Health and Social Care (DHSC) ignores a growing body of evidence on nature’s health benefits. It is striking that other government departments have policy initiatives that directly or indirectly recognise or promote the role of nature and environment in human wellbeing. For example, a core strategy of the Department for Environment, Food and Rural Affairs is to protect and restore the country’s biodiversity. The Department for Culture, Media and Sport is focused on increasing participation in sport and outdoor activity. The Ministry of Housing, Communities and Local Government leads efforts to increase access to green space. The Department of Education recognises green space and nature as essential for children’s education and development. The Department of Transport emphasises healthy transport options such as cycling and walking.

A useful step would be for the Cabinet Office, Chief Medical Officer’s department, or other central coordinating function to ensure that these initiatives are understood as interrelated and mutually reinforcing. Examining international experiences can provide valuable insights for integrating nature and biodiversity into national policy. For example, several European countries have aligned national policies with the EU Biodiversity Strategy for 2030, which aims to protect nature and reverse the degradation of ecosystems [72]. Finland has integrated protection of biodiversity and nature-based wellbeing into its broader health and sustainability agenda [73]. To support effective implementation, the Finnish government has prioritised ongoing monitoring and evaluation of these initiatives. Lessons from these international approaches may help inform the integration of nature and biodiversity within health policy in other countries. This is an organisational and cultural shift, one outcome of which could be a more effective, cost-effective, and imaginative implementation of the 10 Year Health Plan.

2 *A Nature Champion*

Linked to the first step, the Government could appoint a high-profile Champion for integrating nature into health and social care policy.

For example, in his 2024 annual report, the Chief Medical Officer focused on health in our cities, and cited limited access to healthy food, growing obesity, high air pollution, and lack of biodiverse green space as factors contributing to poor health that were solvable with good policy.

An analysis, speech, and other promotion by a respected health professional of these issues could throw light on the lack of joined-up thinking and scope to do better under the 10 Year Health Plan. The Champion could remain engaged in these issues within the DHSC’s strategic delivery process.

3 *A Nature and Health Working Group*

Linked to the previous steps, a third step could be the establishment of a senior officials and wider stakeholders’ Working Group, led by the DHSC, to agree a coordinated approach to nature-based opportunities for health care. This group could monitor and assess research and evidence, review existing initiatives, identify opportunities for integrating nature-based approaches into strategy and policy, and provide recommendations to Ministers and senior officials.

Scotland has pioneered initiatives in this space—some outlined in this paper—bringing together different stakeholders. For example, the Natural Health Service concept, bringing together Public Health Scotland, Scottish Forestry, local authorities, and local partners, launched four Green Health Partnerships, among other initiatives. NHS England should have and demonstrate a comprehensive understanding of nature-based prevention and care research, evidence, and potential opportunities, and consider systematically integrating them into its prevention and community care strategies.

The Group could identify how different initiatives relate to and support each other including quick wins: activities and policies that are already underway and demonstrating interesting results. For example, there has been a resurgence in cycling [74], open-water swimming, and community food growing in recent years, with increasing evidence around the mental health benefits. This could lead to a more proactive public information campaign or other programming to promote the health benefits of these or similar activities.

4 *Economic assessment*

If preventative and community-based goals for nature-based mental healthcare and prevention are agreed and implemented by different stakeholders and government departments, this is highly likely to lead to substantial cost savings.

An official analysis of the economic and financial gains by the economic and finance ministry, office of budget responsibility or national audit office could provide helpful momentum and organisational and culture change. Cost-benefit analysis should be conducted to demonstrate the value of any nature-based intervention, including their potential to reduce long-term healthcare costs and wider fiscal burdens. This could provide governments and funding bodies with the evidence needed to demonstrate that preventive interventions generate greater economic returns than reactive approaches. Furthermore, cost–benefit analyses would enable policymakers to assess the fiscal sustainability of nature-based interventions, identify the most cost-effective strategies, and avoid allocating resources to initiatives that yield limited public health or environmental benefits. Such analyses should also consider the wider co-benefits of nature-based interventions, including improvements in physical health, biodiversity, climate resilience, and social cohesion.

## Conclusion

Our natural environment and health system face huge challenges. Historically, we have treated them separately, with policies on our natural environment rarely integrating with health and social care policies. But this ignores how deeply connected we are to our environment: nature provides invaluable support and restoration for our mental and physical health.

Furthermore, we have adopted an extractive mindset which treats the natural world as an unlimited resource for human benefit; yet the continued depletion of nature ultimately undermines our own health and wellbeing.

This paper suggests that by recognising the interdependencies between our mental health and our natural environment, we can improve both. This is timely, as both areas are under intensive policy scrutiny in the UK, in what is a tight fiscal environment where cost-effective, long-term, preventive, and community-based measures are at a premium.

By seizing the opportunities outlined here, we could achieve real changes in population mental health and the richness of our natural environment. The 10 Year Health Plan marks a unique opportunity to embed this holistic approach into government policy, placing nature at the heart of our future health.
